# Senomorphic Activity of a Novel Standardized Propolis Extract in Human Dermal Fibroblasts: Molecular Insights Into Clinically Proven Anti‐Wrinkle Efficacy

**DOI:** 10.1111/jocd.70689

**Published:** 2026-03-19

**Authors:** Božo Radić, Jelena Šuran

**Affiliations:** ^1^ Apiotix Technologies d.o.o. Split Croatia

**Keywords:** CDK4, cellular senescence, human dermal fibroblasts, IL‐6, inflammaging, propolis, SASP, senomorphic, standardized extract

## Abstract

**Background:**

We recently demonstrated in a randomized controlled trial (RCT) that a Standardized Propolis Extract (SPE), produced via a patented non‐alcoholic PEG 400/lecithin process, achieves significant clinical anti‐wrinkle efficacy (34% wrinkle depth reduction). The present study investigates the underlying molecular mechanisms, specifically its potential senomorphic activity—the ability to modulate the Senescence‐Associated Secretory Phenotype (SASP) without inducing cell death.

**Objective:**

To evaluate the senomorphic activity of this chemically defined SPE (standardized to 1318.43 μg/g total phenolic markers) in an in vitro model of oxidative stress‐induced senescence, providing molecular insights into its clinically observed anti‐aging effects.

**Methods:**

Human Dermal Fibroblasts (HDFs) were pre‐treated with SPE (0.01%, 0.05%) or Rapamycin (3 μM, reference senomorphic control). Senescence was induced via a validated stress‐induced premature senescence (SIPS) protocol (200 μM H_2_O_2_, 2 h). Gene expression for senescence markers (CDKN2A/p16, CDKN1A/p21), SASP cytokines (IL‐6, IL‐8), and cell cycle regulators (CDK4, CDK2, CCNE1) was quantified by qPCR. An exploratory study on Mesenchymal Stem Cells (MSCs) assessed SA‐β‐galactosidase activity qualitatively.

**Results:**

The 0.05% SPE demonstrated potent senomorphic activity, significantly suppressing the key SASP marker IL‐6 (FC: −7.78, *p* = 0.003)—comparable to the Rapamycin control (FC: −8.1, *p* = 0.003). Uniquely, SPE induced transcriptional upregulation of CDK4 (FC: +6.71, *p* = 0.002) and CDKN1A/p21 (FC: +2.33, *p* = 0.005), effects not observed with Rapamycin. In exploratory MSC experiments, SPE qualitatively reduced SA‐β‐gal staining.

**Conclusion:**

This first‐in‐class standardized propolis extract demonstrates distinct senomorphic activity, suppressing the inflammatory SASP (IL‐6) while inducing transcriptional modulation of pro‐regenerative pathways (CDK4). These molecular findings provide mechanistic insights consistent with the extract's clinically proven anti‐wrinkle efficacy, supporting its positioning as an evidence‐based active ingredient for dermo‐cosmetic formulations targeting inflammaging.

## 1 Introduction

Skin aging is driven by both intrinsic chronological factors and extrinsic environmental insults, particularly oxidative stress from solar radiation and pollution. A central mechanism underlying extrinsic aging is the induction of cellular senescence in dermal cell populations, especially Human Dermal Fibroblasts (HDFs) [1, 2, 3].

Cellular senescence is characterized by permanent cell cycle arrest, mediated primarily by the tumor suppressors CDKN2A (p16) and CDKN1A (p21) [4]. While senescence serves as a tumor‐suppressive mechanism, the accumulation of senescent cells in aging tissues is pathological. Critically, senescent cells adopt a pro‐inflammatory Senescence‐Associated Secretory Phenotype (SASP), secreting cytokines (IL‐6, IL‐8), chemokines, and matrix metalloproteinases (MMPs) that drive chronic low‐grade inflammation (“inflammaging”) and extracellular matrix degradation [3]. Targeting the SASP—rather than eliminating senescent cells—represents a promising therapeutic strategy. Agents that modulate the SASP without inducing cell death are termed *senomorphics*, in contrast to *senolytics*, which selectively kill senescent cells [5].

Propolis, a resinous bee product, possesses well‐documented antioxidant and anti‐inflammatory properties attributed to its phenolic acid content, including p‐coumaric acid, trans‐ferulic acid, caffeic acid, and Caffeic Acid Phenethyl Ester (CAPE) [6, 7]. These compounds modulate key signaling pathways including Nrf2 and NF‐κB [7, 8]. However, the therapeutic application of propolis has been hindered by compositional inconsistency inherent to traditional ethanol‐based extraction methods, which also introduce solvent‐related limitations and high beeswax content causing formulation instability [9, 10].

To overcome these limitations, we developed a first‐in‐class Standardized Propolis Extract (SPE) using a proprietary, non‐alcoholic extraction system based on PEG 400 and lecithin. This chemoselective process, protected by an internationally granted patent (WO2020169425A1) [11], yields an extract with a defined and reproducible chemical profile—a critical differentiator from generic propolis preparations. The patent specifically protects this unique solvent composition and the resulting standardized phenolic fingerprint, which cannot be achieved with conventional extraction methods.

The clinical efficacy of this exact SPE has already been established. In a recent 28‐day, double‐blind, randomized controlled trial (RCT) published in this journal, we demonstrated that a 3% SPE formulation achieved a 34% reduction in wrinkle depth compared to baseline [12]. That study also showed, via BioMAP profiling, that the extract suppresses MMP‐1 and MMP‐9 while upregulating Collagen III. However, the fundamental cellular mechanisms underlying these clinical and biochemical effects—particularly regarding senescence biology—remained unexplored.

Therefore, the objective of this study was to investigate whether this chemically defined SPE acts as a senomorphic agent, specifically examining its effects on SASP cytokine expression and cell cycle regulatory gene transcription in a validated in vitro model of oxidative stress‐induced senescence.

## 2 Materials and Methods

### 2.1 Preparation and Chemical Characterization of the Standardized Propolis Extract (SPE)

The SPE (Lot #102024) was prepared using the proprietary extraction method described in patent WO2020169425A1 [11]. The extraction solvent was formulated by mixing Polyethylene Glycol 400 (PEG 400) and soy lecithin in a 97:3 w/w ratio. Milled, crude propolis was macerated in this solvent system for 72 h at room temperature. After filtration to remove insoluble residue, the final liquid SPE was obtained with a drug‐to‐extract ratio (DER) of approximately 1:4.

#### 2.1.1 Chemical Standardization

**FIGURE 1:**
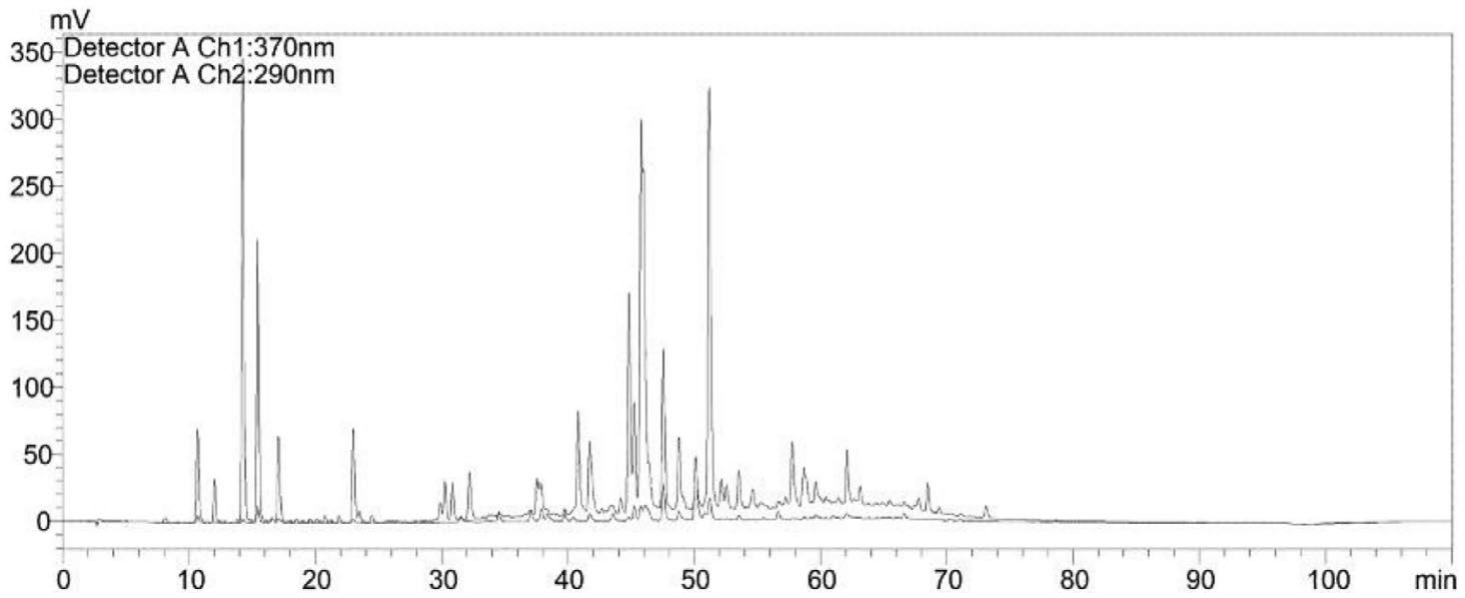
HPLC chromatogram and chemical standardization of SPE. Representative chromatogram showing the four standardized phenolic markers: (1) caffeic acid (218.08 μg/g), (2) p‐coumaric acid (524.74 μg/g), (3) trans‐ferulic acid (544.06 μg/g), and (4) CAPE (31.55 μg/g). Total standardized content: 1318.43 μg/g. The phenolic profile is dominated by the hydroxycinnamic acids trans‐ferulic and p‐coumaric, consistent with our published characterization [12].

The SPE was characterized by High‐Performance Liquid Chromatography (HPLC) according to the validated method described in our previous publication [11]. The extract is standardized to a total concentration of 1318.43 μg/g for the four key phenolic markers: caffeic acid (218.08 μg/g), p‐coumaric acid (524.74 μg/g), trans‐ferulic acid (544.06 μg/g), and CAPE (31.55 μg/g) (Figure 1, table 1 in Reference [12]). This quantified, reproducible chemical profile—with trans‐ferulic and p‐coumaric acids as the dominant components—is a defining characteristic of the SPE and distinguishes it from non‐standardized propolis preparations.

### 2.2 Human Dermal Fibroblast (HDF) Senescence Model

#### 2.2.1 Cell Culture

Normal human dermal fibroblasts from an adult donor (HDF 23yr, Lifeline Cell Technology, Cat.# FC‐0024, Lot# 3869, Passage 8) were cultured in DMEM supplemented with 10% FBS at 37°C and 5% CO_2_.

#### 2.2.2 Senescence Induction and Treatment Protocol

A stress‐induced premature senescence (SIPS) model was established following validated protocols [2, 14, 15]. Cells were pre‐treated for 24 h with SPE (final concentrations: 0.01%, 0.05%, or 0.15%), Rapamycin (3 μM, reference senomorphic control), or vehicle control (0.5% PEG 400). Senescence was induced by exposing cells to 200 μM H_2_O_2_ in PBS for 2 h [13, 15]. Following exposure, the H_2_O_2_‐containing medium was removed, replaced with fresh growth medium containing the respective treatments, and cells were incubated for an additional 6 days to allow the senescent phenotype to develop [14].

### 2.3 Exploratory Mesenchymal Stem Cell (MSC) Study

As an exploratory, qualitative assessment, normal human adipose‐derived MSCs (ATCC, Cat.# PCS‐500‐011) were cultured in MSC basal medium. To induce stress, cells were placed in starvation medium 1 day prior to treatment with 0.01% SPE. After 7 days, cells were assessed qualitatively for SA‐β‐galactosidase activity.

### 2.4 Cytotoxicity Assessment and Biomarker Analysis

#### 2.4.1 Cytotoxicity

Cell viability was assessed using LDH release [16] and sulforhodamine B (SRB) assays. Conditions exhibiting > 15% cytotoxicity (*p* < 0.05) versus non‐treated controls were excluded from gene expression analysis.

#### 2.4.2 SA‐β‐Galactosidase Staining

**FIGURE 2:**
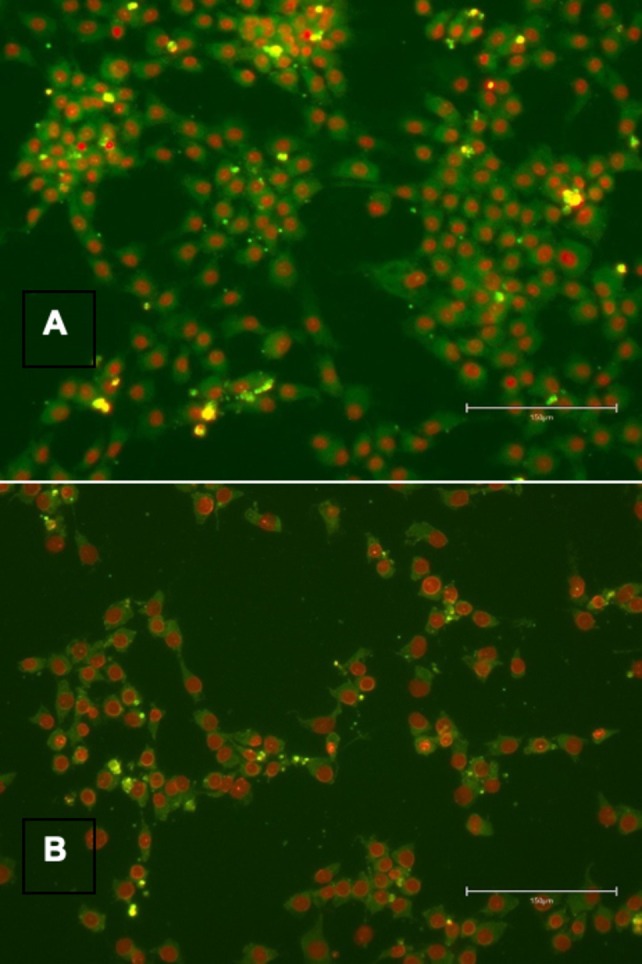
Exploratory SA‐β‐galactosidase assessment in MSCs. Qualitative fluorescence microscopy of MSCs under starvation stress. (A) Vehicle control showing high SA‐β‐gal activity (green) and senescent morphology. (B) 0.01% SPE‐treated cells showing qualitatively reduced SA‐β‐gal staining. These exploratory findings support further quantitative investigation.

In the exploratory MSC study, SA‐β‐gal activity was qualitatively assessed using a fluorescent detection kit (Dojindo, Cat.# DG04) and visualized by fluorescence microscopy (Figure 2).

#### 2.4.3 Gene Expression Analysis (qPCR)

Total RNA was extracted from HDFs using the RNeasy Mini kit (Qiagen). cDNA was synthesized using the High‐Capacity RNA‐to‐cDNA Kit (ThermoFisher, Cat. #4387406). Real‐time quantitative PCR was performed on a Bio‐Rad C1000 Touch System with CFX384 optical module using Forget‐Me‐Not EvaGreen qPCR Master Mix (Biotium, Lot #23F1213) and commercially validated primer sets (RealTimePrimers, Elkins Park, PA). The gene panel included: CDKN1A (p21), CDKN2A (p16), CCNE1, CDK2, CDK4, IL6, and IL8. Expression was normalized to HPRT1, which demonstrated stable expression (FC: 1.0) under our oxidative stress conditions.

### 2.5 Statistical Analysis

Gene expression was quantified using the Efficiency ΔΔCt method. All experiments were performed in triplicate. Statistical significance was determined by two‐tailed *t*‐test, with *p* < 0.05 considered significant.

## 3 Results

### 3.1 Dose Selection Based on Cytotoxicity Profiling

The 0.15% SPE concentration exhibited excessive cytotoxicity (> 75% reduction in cell number) and was excluded. The 0.05% concentration was selected as the Maximum Tolerated Dose (MTD), producing a cytostatic effect comparable to the reference senomorphic agent Rapamycin (3 μM), without inducing cell death. The 0.01% concentration was confirmed as non‐cytotoxic.

### 3.2 Validation of the H_2_O_2_‐Induced Senescence Model

The 200 μM H_2_O_2_ treatment successfully induced a robust senescent and inflammatory phenotype in vehicle‐control HDFs. Compared to non‐stressed controls, H_2_O_2_‐exposed cells exhibited significant upregulation of CDKN2A/p16 (FC: +5.8, *p* < 0.001) and IL6 (FC: +19.3, *p* = 0.003), confirming successful senescence induction with SASP activation (Table 1).

**TABLE 1:** Gene expression analysis in H_2_O_2_‐stressed HDFs.

Gene marker	Protein function	Condition 1: H_2_O_2_ stress (vs. no stress)	Condition 2: Rapamycin 3 μM (vs. stressed control)	Condition 3: SPE 0.05% (vs. stressed control)	Condition 4: SPE 0.01% (vs. stressed control)
		Fold change (*p*‐value)	Fold change (*p*‐value)	Fold change (*p*‐value)	Fold change (*p*‐value)
CDKN2A	p16 (Irreversible senescence)	**+5.8 (*p* = 0.000)**	**−2.2 (*p* = 0.002)**	+1.05 (*p* = 0.677)	**+16.49 (*p* = 0.041)**
IL6	SASP (chronic inflammation)	**+19.3 (*p* = 0.003)**	**−8.1 (*p* = 0.003)**	**−7.78 (*p* = 0.003)**	**−2.03 (*p* = 0.021)**
CDK4	G1‐S phase progression	NM	NM	**+6.71 (*p* = 0.002)**	**+2.87 (*p* = 0.020)**
CDKN1A	p21 (temporary arrest/repair)	NM	NM	**+2.33 (*p* = 0.005)**	NM
IL8	SASP (localized repair)	NM	NM	**+3.07 (*p* = 0.023)**	NM
CDK2	DNA replication	NM	NM	**+1.17 (*p* = 0.037)**	NM
CCNE1	G1/S transition	NM	NM	−1.10 (*p* = 0.363)	NM
HPRT1	Housekeeping gene	—	1.0	1.0	1.0

*Note:* qPCR results from HDFs exposed to 200 μM H_2_O_2_ and subsequently treated for 6 days with controls or SPE. All comparisons are made relative to the H_2_O_2_‐stressed vehicle‐treated control group. NM (not modulated/not measured) indicates the change was not statistically significant (*p* < 0.05) or not measured under that specific condition. Values in bold indicate statistically significant differences (*p* < 0.05) compared to the H_2_O_2_‐only stressed control and in the groups treated with the Standardized Propolis Extract (SPE).

### 3.3 Senomorphic Activity: SASP Modulation

Effects were measured relative to the H_2_O_2_‐stressed vehicle control (Table 1).

#### 3.3.1 Rapamycin (Reference Senomorphic Control)

As expected, Rapamycin significantly suppressed both CDKN2A/p16 (FC: −2.2, *p* = 0.002) and IL6 (FC: −8.1, *p* = 0.003), confirming its established senomorphic activity.

#### 3.3.2 SPE 0.05%

This concentration demonstrated potent senomorphic activity with a significant suppression of IL6 (FC: −7.78, *p* = 0.003), comparable in magnitude to Rapamycin. Interestingly, IL8 was moderately upregulated (FC: +3.07, *p* = 0.023). Notably, SPE did not significantly alter CDKN2A/p16 expression (FC: +1.05, *p* = 0.677), suggesting a mechanism distinct from direct cell cycle inhibitor suppression.

#### 3.3.3 SPE 0.01%

The lower concentration showed significant, albeit weaker, IL6 suppression (FC: −2.03, *p* = 0.021), supporting a dose‐dependent senomorphic effect.

### 3.4 Transcriptional Modulation of Cell Cycle Regulators

**FIGURE 3:**
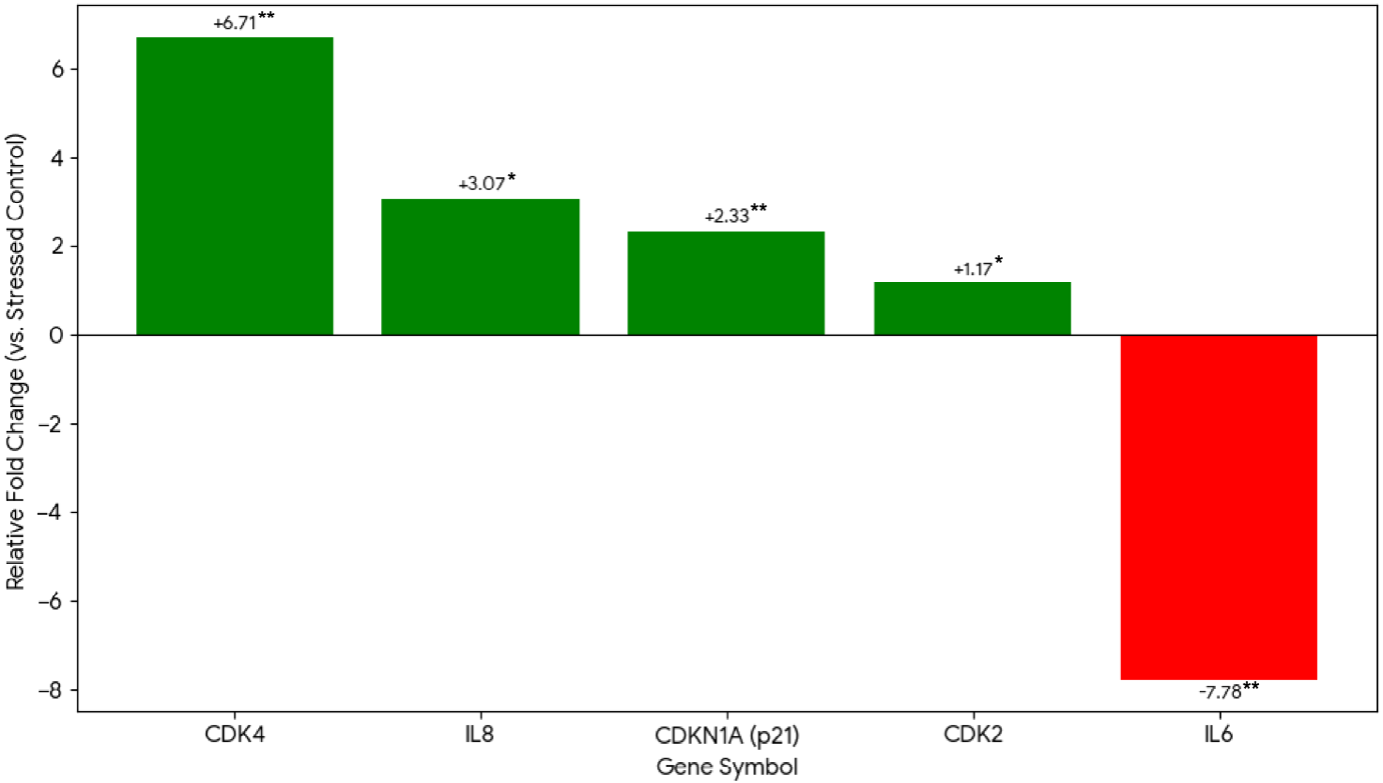
Transcriptional profile of 0.05% SPE in stressed HDFs. Fold change in gene expression demonstrating the dual senomorphic activity: IL6 suppression (FC: −7.78) concurrent with CDK4 upregulation (FC: +6.71). Data: Mean ± SD (*n* = 3), *p < 0.05, **p < 0.01.

The most distinctive finding was the differential effect of SPE versus Rapamycin on cell cycle gene transcription (Table 1; Figure 3).

#### 3.4.1 Rapamycin

Consistent with its mechanism as a quiescence inducer, Rapamycin had no significant effect on CDK4, CDK2, CCNE1, or CDKN1A/p21.

#### 3.4.2 SPE 0.05%

In contrast, SPE induced significant transcriptional upregulation of CDK4 (FC: +6.71, *p* = 0.002) and CDKN1A/p21 (FC: +2.33, *p* = 0.005). CDK2 showed modest upregulation (FC: +1.17, *p* = 0.037), while CCNE1 was unchanged.

#### 3.4.3 SPE 0.01%

Dose‐dependent CDK4 upregulation was confirmed (FC: +2.87, *p* = 0.020).

### 3.5 Exploratory MSC Study: Qualitative SA‐β‐Gal Assessment

In the exploratory MSC study under starvation stress, 0.01% SPE did not produce statistically significant changes in stemness gene expression (OCT4, SOX2, WNT2). However, qualitative fluorescence microscopy revealed a visible reduction in SA‐β‐gal staining (green fluorescence) and fewer cells displaying the characteristic enlarged, flattened senescent morphology in the SPE‐treated group compared to vehicle controls (Figure 2). These exploratory observations warrant further quantitative investigation.

## 4 Discussion

This study provides the first evidence that a chemically defined, standardized propolis extract acts as a senomorphic agent in human dermal fibroblasts. The key finding is that SPE potently suppresses IL‐6—a central mediator of the SASP and inflammaging [1]—with efficacy comparable to the established senomorphic agent Rapamycin, while simultaneously inducing distinct transcriptional changes in cell cycle regulatory genes.

### 4.1 Distinct Senomorphic Mechanism

The transcriptional profile induced by SPE differs fundamentally from Rapamycin. While both suppress IL‐6, only SPE upregulates CDK4 and p21 transcription. This pattern suggests that SPE does not simply induce cellular quiescence (the Rapamycin mechanism), but may favor a p21‐mediated temporary arrest permissive for DNA damage repair [17, 18]. The concurrent CDK4 upregulation is intriguing, as CDK4 is essential for G1‐S phase progression. We hypothesize that this transcriptional priming may facilitate cell cycle re‐entry following resolution of oxidative damage—a “pro‐recovery” state rather than terminal quiescence. Importantly, we emphasize that these are *transcriptional observations*; functional validation of cell cycle dynamics via flow cytometry and protein‐level confirmation via Western blot are required to substantiate this hypothesis.

### 4.2 The Importance of Standardization

A critical distinction of this work is the use of a chemically defined, standardized extract. The patented PEG 400/lecithin extraction process [11] yields a reproducible phenolic profile (1318.43 μg/g total markers), enabling consistent biological activity across batches—a prerequisite for both scientific reproducibility and commercial application. This standardization differentiates our SPE from the variable compositions characteristic of conventional ethanol extracts and is the basis for the granted international patent protection.

### 4.3 Molecular Insights Consistent With Clinical Efficacy

This mechanistic study complements our published RCT demonstrating 34% wrinkle depth reduction with this same SPE [12]. The BioMAP data from that study showed MMP suppression and Collagen III upregulation—effects on the extracellular matrix. The present findings add a complementary dimension: senomorphic activity targeting the *cellular* drivers of inflammaging. Together, these data suggest a multi‐pathway mechanism: (1) structural matrix support via MMP inhibition and collagen synthesis [12], and (2) inflammaging mitigation via SASP suppression (this study). While we cannot establish direct causality between in vitro gene expression changes and clinical outcomes, these molecular findings provide mechanistic insights consistent with the observed clinical anti‐wrinkle efficacy.

### 4.4 Limitations

Several limitations warrant consideration. First, this study reports transcriptional (mRNA) changes; protein‐level validation (Western blot, ELISA) is needed to confirm functional pathway activation. Second, the MSC data are exploratory and qualitative; quantitative SA‐β‐gal assays and functional assessments are required. Third, the 0.01% dose paradoxically upregulated p16 in HDFs, suggesting a hormetic dose–response [19] that requires further characterization. Similarly, the moderate upregulation of IL‐8 (+3.07) observed with 0.05% SPE requires further functional characterization, as IL‐8 is known to play dual roles in both SASP‐driven inflammation and pro‐regenerative tissue repair pathways [20]. Finally, translation from in vitro models to in vivo skin biology involves complex factors not captured in monoculture systems.

## 5 Conclusion

This first‐in‐class Standardized Propolis Extract, defined by its patented extraction process and quantified phenolic content (1318.43 μg/g), demonstrates senomorphic activity in oxidatively stressed human dermal fibroblasts. The 0.05% concentration potently suppresses IL‐6 (the key SASP cytokine) while inducing transcriptional upregulation of CDK4 and p21—a profile distinct from the quiescence‐inducing Rapamycin. These molecular findings provide mechanistic insights consistent with the extract's clinically proven anti‐wrinkle efficacy [12], supporting its positioning as a scientifically validated active ingredient for evidence‐based dermo‐cosmetic formulations targeting skin inflammaging.

## Ethical Statement

The authors confirm that the ethical policies of the journal have been adhered to. Ethical approval was not required as this study was conducted in vitro using commercially available, certified human cell lines.

## Consent

Informed consent was obtained from original tissue donors by the commercial vendors (Lifeline Cell Technology and ATCC), in accordance with the Declaration of Helsinki.

## Conflicts of Interest

Božo Radić and Jelena Šuran are employees and shareholders of Apiotix Technologies d.o.o., the company that holds the internationally granted patent (WO/2020/169425, with patents granted in the European Union [EP2020706145], United States [US17430567], China [CN202080015084.X], India [IN202117037021], Eurasia [EA202192022], and Mexico [MX/a/2021/009877]) for the standardized propolis extract evaluated in this study.

## Data Availability

The data that support the findings of this study are available from the corresponding author upon reasonable request.

## References

[1] O. Toussaint , E. E. Medrano , and T. von Zglinicki , “Cellular and Molecular Mechanisms of Stress‐Induced Premature Senescence (SIPS),” Experimental Gerontology 35, no. 8 (2000): 927–945. 11121681 10.1016/s0531-5565(00)00180-7

[2] T. Y. McCarty and C. J. Kearney , “Human Dermal Fibroblast Senescence in Response to Single and Recurring Oxidative Stress,” Frontiers in Aging 6 (2025): 1504977. 40225319 10.3389/fragi.2025.1504977 PMC11985536

[3] C. Pan , H. Lang , T. Zhang , et al., “Conditioned Medium Derived from Human Amniotic Stem Cells Delays H_2_O_2_‐Induced Premature Senescence in Human Dermal Fibroblasts,” International Journal of Molecular Medicine 44, no. 5 (2019): 1629–1640. 31545472 10.3892/ijmm.2019.4346 PMC6777671

[4] J. Yan , S. Chen , Z. Yi , et al., “The Role of p21 in Cellular Senescence and Aging‐Related Diseases,” Molecules and Cells 47, no. 11 (2024): 100113. 39304134 10.1016/j.mocell.2024.100113 PMC11564947

[5] J. L. Kirkland and T. Tchkonia , “Senolytic Drugs: From Discovery to Translation,” Journal of Internal Medicine 288, no. 5 (2020): 518–536. 32686219 10.1111/joim.13141 PMC7405395

[6] J. Šuran , I. Cepanec , T. Mašek , et al., “Propolis Extract and Its Bioactive Compounds—From Traditional to Modern Extraction Technologies,” Molecules 26, no. 10 (2021): 2930. 34069165 10.3390/molecules26102930 PMC8156449

[7] C. Scorza , V. Goncalves , J. Finsterer , F. Scorza , and F. Fonseca , “Exploring the Prospective Role of Propolis in Modifying Aging Hallmarks,” Cells 13, no. 5 (2024): 390. 38474354 10.3390/cells13050390 PMC10930781

[8] D. H. Kim , J. H. Auh , J. Oh , et al., “Propolis Suppresses UV‐Induced Photoaging in Human Skin Through Directly Targeting Phosphoinositide 3‐Kinase,” Nutrients 12, no. 12 (2020): 3790. 33322005 10.3390/nu12123790 PMC7764066

[9] Z. Sosnowski , Method for Extracting Propolis and Water Soluble Dry Propolis Powder (1983). US Patent 4382886.

[10] T. Tsukada and T. Nakajima , “Production of Water‐Soluble Propolis Pharmaceutical Preparation,” (1993), JP Patent JPH05957A.

[11] S. Radić , B. Radić , and J. Šuran , “Liquid Propolis Extract, Its Formulation and Use Thereof,” (2020), WO Patent WO2020169425A1.

[12] B. Radić , S. Radić , T. Mašek , and J. Šuran , “Anti‐Wrinkle Efficacy of Standardized Phenolic Acids Polymer Extract (PAPE) From Propolis: Implications for Antiaging and Skin Health,” Journal of Cosmetic Dermatology 23 (2024): 3372–3381. 38943252 10.1111/jocd.16405 PMC13648926

[13] Q. Chen and B. N. Ames , “Senescence‐Like Growth Arrest Induced by Hydrogen Peroxide in Human Diploid Fibroblast F65 Cells,” Proceedings of the National Academy of Sciences of the United States of America 91 (1994): 4130–4134. 8183882 10.1073/pnas.91.10.4130 PMC43738

[14] M. Gerasymchuk , G. I. Robinson , O. Kovalchuk , and I. Kovalchuk , “Modeling of the Senescence‐Associated Phenotype in Human Skin Fibroblasts,” International Journal of Molecular Sciences 23, no. 13 (2022): 7124. 35806127 10.3390/ijms23137124 PMC9266450

[15] J. Chen , S. E. Ozanne , and C. N. Hales , “Methods of Cellular Senescence Induction Using Oxidative Stress,” Methods in Molecular Biology 371 (2007): 179–189. 17634582 10.1007/978-1-59745-361-5_14

[16] T. Decker and M. L. Lohmann‐Matthes , “A Quick and Simple Method for the Quantitation of Lactate Dehydrogenase Release in Measurements of Cellular Cytotoxicity and Tumor Necrosis Factor (TNF) Activity,” Journal of Immunological Methods 115, no. 1 (1988): 61–69. 3192948 10.1016/0022-1759(88)90310-9

[17] O. V. Leontieva and M. V. Blagosklonny , “CDK4/6‐Inhibiting Drug Substitutes for p21 and p16 in Senescence,” Cell Cycle 12, no. 18 (2013): 3063–3069. 23974099 10.4161/cc.26130 PMC3875680

[18] C. Capparelli , B. Chiavarina , D. Whitaker‐Menezes , et al., “CDK Inhibitors (p16/p19/p21) Induce Senescence and Autophagy in Cancer‐Associated Fibroblasts, ‘Fueling’ Tumor Growth via Paracrine Interactions, Without an Increase in Neo‐Angiogenesis,” Cell Cycle 11, no. 19 (2012): 3599–3610. 22935696 10.4161/cc.21884 PMC3478311

[19] E. J. Calabrese and L. A. Baldwin , “Defining Hormesis,” Human & Experimental Toxicology 21, no. 2 (2002): 91–97. 12102503 10.1191/0960327102ht217oa

[20] D. J. J. Waugh and C. Wilson , “The Interleukin‐8 Pathway in Cancer,” Clinical Cancer Research 14, no. 21 (2008): 6735–6741. 18980965 10.1158/1078-0432.CCR-07-4843

